# BEYOND BATTERIES: PORTABLE HYDROGEN FUEL CELLS

**DOI:** 10.1289/ehp.115-a38

**Published:** 2007-01

**Authors:** Carol Potera

Mention hydrogen fuel cells, and most people envision hydrogen-powered cars as an alternative to the gas-guzzling and polluting internal combustion engine. In fact, many much smaller applications also could benefit from this nonpolluting technology. However, despite the billions of dollars being poured into the research and development of hydrogen fuel cells, few products have been commercialized.

To jump-start the hydrogen fuel cell economy, Larry Bawden and Lee Arikara cofounded Jadoo Power Systems in 2001. While demonstrating their technology at a convention in 2002, an observer remarked that the creation of electricity from hydrogen seemed like magic. The company’s name grew from that comment—Jadoo means “magic” in Hindi. “We wanted a nontechnical name to brand ourselves as a provider of solutions, not another technical house,” says Bawden, Jadoo’s president and CEO.

A small company with 40 workers, Jadoo searches for applications that require 100–500 watts of power (a hydrogen car, for comparison, needs 50,000–100,000 watts to give it the pep of a typical gasoline-powered car). Jadoo first targeted the cumbersome and inefficient batteries hauled around by television news crews. Television camera operators typically carry three rechargeable “brick” batteries, each weighing about six pounds and costing $500. An additional battery charger costs $1,500. According to Arikara, Jadoo’s vice president of business development, television stations spend an average of $3,500 per camera to outfit them with batteries. Further, while swapping out a dead video battery, all power stops, and critical film footage can be lost.

This made the broadcast industry an ideal niche market to demonstrate that hydrogen fuel cells could do the job better and cheaper. The experts at Jadoo designed and manufactured a 100-watt hydrogen fuel cell system, called N-Gen™, to fit professional video cameras. N-Gen weighs five pounds, and it’s fueled by a two-pound hydrogen-filled canister called N-Stor™. A small reservoir retains enough hydrogen to power the cameras for up to 30 seconds while replacing an empty hydrogen canister, allowing the camera to run continuously.

Rechargeable batteries lose capacity with each recharge and eventually are discarded, adding to llandfill contamination with metals such as nickel and cadmium. But fuel cells do not discharge and degrade over time. Theoretically, the metal hydride should not wear out, though in practical use it could deteriorate. The company recommends that customers return the canisters every five years so the metal hydride can be checked.

A new rechargeable brick battery runs for about two hours, whereas N-Gen can last four to five hours before needing a refill of hydrogen. A brick battery takes about six hours to recharge, up to six times longer than it takes to refill an N-Stor canister. In short, television camera operators can replace three inefficient six-pound brick batteries with one highly efficient N-Gen system at a total of seven pounds. Moreover, at $2,050, the N-Gen portable power system costs about one-third less than three brick batteries and a battery charger.

The CBS affiliate in Sacramento, California, is testing the Jadoo system. “We’ve had zero problems, and it delivers smooth, steady voltage far longer than any battery pack,” says Kalo Alexandra, remote systems engineer. He’d like Jadoo to reduce the size of the fuel cell and hydrogen canister and add hookups for other equipment like lights and microphones. Overall, the Jadoo system “gives every indication of sounding the death knell for brick batteries in the broadcast industry,” Alexandra says.

Video engineer Dave Titchenal adopted Jadoo’s technology for his video production company in Modesto, California. His crews set up cameras and video projection equipment in large auditoriums and football stadiums, connected to outlets by hundreds of yards of taped-down electrical cords—which feels like miles when you’re on your knees taping it down. He’s replaced all those cords with Jadoo fuel cells. “It’s fabulous to know that you don’t have to be tied to a power grid,” says Titchenal.

## How It Works

“The basic difference between a battery and a fuel cell,” Bawden notes, “is that a battery stores electrons, but our fuel cells make electrons.” Inside a fuel cell, hydrogen and oxygen from the air combine to produce electricity and water vapor, which stays confined to the surface of the N-Stor cartridge until it evaporates. No fuel is burned in this electrochemical process, so no polluting by-products (such as carbon dioxide) are emitted. As long as there’s a supply of hydrogen, electricity flows in a fuel cell.

The N-Gen power system measures about 4 inches by 4 inches by 7 inches, and the N-Stor canisters come in two sizes, holding 130 or 360 watt-hours of energy. The smaller canister, which is used on TV cameras, is about the size of a 12-ounce soda can; the larger size is the same diameter but twice as tall. The fuel canister quickly snaps into a port on the N-Gen. A digital display tells how much fuel remains and the amount of power (watts) being used.

Inside the N-Stor canister, metal hydrides soak up the hydrogen gas like a sponge, tripling the amount of hydrogen that can be packed into the same space. Since the hydride absorbs the hydrogen, no compression is required.

The company is exploring alternative ways to store hydrogen, such as sodium borohydride made from borax. A borohydride canister would weigh significantly less than the current metal hydride type. Because sodium borohydride generates hydrogen simply by adding water, it would not need hydrogen gas refills. Customers would “add water to borohydride packs to activate them, just like astronauts make Tang,” says Bawden.

In the meantime, when a canister needs refilling, it is inserted into Jadoo’s FillOne™ station, which hooks up to a hydrogen tank through a hose. The FillOne station calculates and displays the refill time, usually about two hours for a 130 canister or four hours for a 360. A larger FillPoint™ station refills four 130 canisters simultaneously in about an hour. Or users can ship empty N-Stor canisters to Jadoo for refilling.

As fuel cells are becoming accepted at TV stations, most are buying the larger FillPoint at a cost of about $1,700. The Jadoo system uses industrial-grade hydrogen that is easy to buy through local welding supply stores. A “K-cylinder” of compressed hydrogen, enough to fill about 55 to 60 N-Stor 130 canisters, costs around $50.

Because the original N-Gen system was created for the broadcast industry, it contains the same interface found on rechargeable brick batteries to attach it to video cameras. To make N-Gen more versatile, Jadoo has since added a 12-volt direct current interface (like the one that plugs into a car cigarette lighter) and a 110-volt alternating current interface for use with appliances that plug in to the wall. In independent testing of N-Gen for the FuelCellWorks.com news website, the system powered a fan, electric screwdriver, fax machine, electric razor, bug zapper, food mixer, and laptop and desktop computers.

## Expanding Markets

The company’s early success earned Bawden and Arikara the 2006 Ernst & Young Entrepreneur of the Year Award for emerging markets in Northern California. Jadoo’s solutions also caught the attention of the military. Soldiers in the U.S. Army’s Special Operations Command carry 80 pounds of heavy batteries to power field radios that transmit life-and-death messages. Jadoo will be replacing the batteries with a 24-pound fuel cell, and is working to further reduce the weight to 12 pounds. Jadoo’s fuel cells can also power military devices such as robots and unmanned armored vehicles.

Kuchera Defense Systems in Windber, Pennsylvania, uses Jadoo’s fuel cells to power its T2 inspection robots that check for explosives under military and civilian vehicles. Bob Unger, program manager of advanced systems at Kuchera, says Jadoo’s N-Gen system operating on one hydrogen canister extends the life of a T2 robot approximately threefold over the commonly used BA-5590 lithium battery.

“The military is in need of smaller and lighter power sources for portable devices, and Jadoo’s technology ideally suits them,” Unger says. At the new Center for Excellence for Advanced Energy Systems in Manufacturing, funded by the Pennsylvania Energy Development Authority and located at the Kuchera facility, researchers will create environmentally friendly commercial products that integrate Jadoo’s fuel cell systems and other advanced energy devices.

Jadoo recently introduced the XRT system designed for first responders in emergency preparedness situations. The system includes the N-Gen fuel cell and six 360 canisters. Hurricane Katrina highlighted the tragic effects of lost electrical power—batteries powering the radios of first responders died the first day, and there was no electricity to recharge them. The XRT weighs only 50 pounds, yet furnishes the same energy as the 200 pounds’ worth of lithium ion batteries that commonly power emergency equipment. The XRT can power portable radios, laptop computers, emergency lighting, satellite phones, and modems during electrical failures. “Fuel cells with enough hydrogen canisters could have supplied power during the entire time that the power was out in New Orleans,” says Bawden.

In Jadoo’s vision for the future, fuel cells will power home appliances or even entire houses. Moreover, fuel cells could supply steady electricity for residents, travelers, and humanitarian aide workers in regions throughout the world that do not have electricity or a reliable power source.

## Building the Hydrogen Ecosystem

Jadoo was the first, and remains the only, company to receive approval from the U.S. Department of Transportation to ship hydrogen fuel cells as air cargo. Bawden says misconceptions about the safety of hydrogen gas initially made regulators skeptical about the safety of the metal hydrides in the N-Stor canisters. However, after enduring dozens of tests showing that the canisters safely withstood bonfire burning, forced overfills, and being shot with rifles, the N-Stor canister gained legal shipping status.

A lack of understanding about the safety of hydrogen is also “slowing the fuel cell economy down,” Bawden says. As a member of the Hydrogen Technical Advisory Committee, which makes recommendations to the Secretary of Energy, he hopes to educate people and change attitudes and policy. “Propane gas in a grill is more dangerous,” he says.

The trailblazers at Jadoo are spearheading the hydrogen economy by continuing to find niche markets for portable fuel cells to prove their commercial feasibility. Experts generally agree that although large-scale environmental gains are not immediate, they will follow when fuel cells are widely accepted. Jadoo’s specialty applications “will drive the price point down, making hydrogen technology commonplace,” predicts Bawden.

“We’re building the ecosystem for the hydrogen industry,” says Arikara. In fuel cell parlance, “ecosystem” refers to the many potential players, such as electronics and valve manufacturers, who are sitting on the sidelines and watching the technology. “They want to see commercialization to ensure a return on their investment in parts, tools, and equipment,” Arikara explains. As more companies become involved, he adds, “prices will become competitive, and the technology will make significant environmental impacts.”

The portable fuel cell market “doesn’t get much attention, but it’s an enabling sector with products available today that are paving the way for consumers to accept hydrogen vehicles,” says Patrick Serfass, director of technology and program development at the National Hydrogen Association, a trade organization. With reduced use of fossil fuels and release of hazardous emissions, Serfass says the widespread acceptance of fuel cells “will bring benefits to the environment, energy security, and economic growth.”

## Figures and Tables

**Figure:**
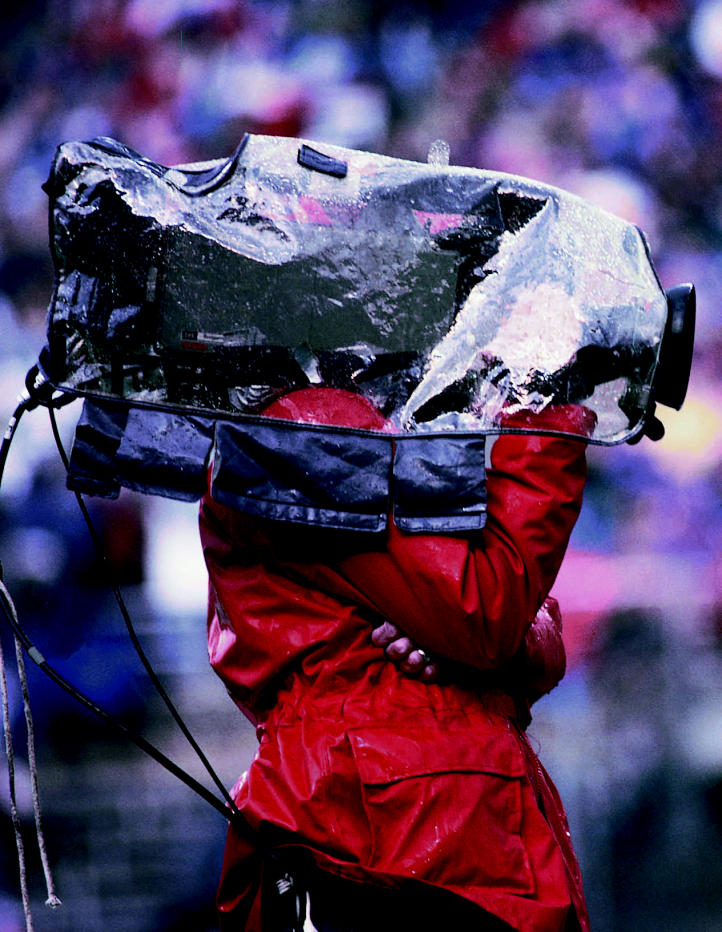


**Figure:**
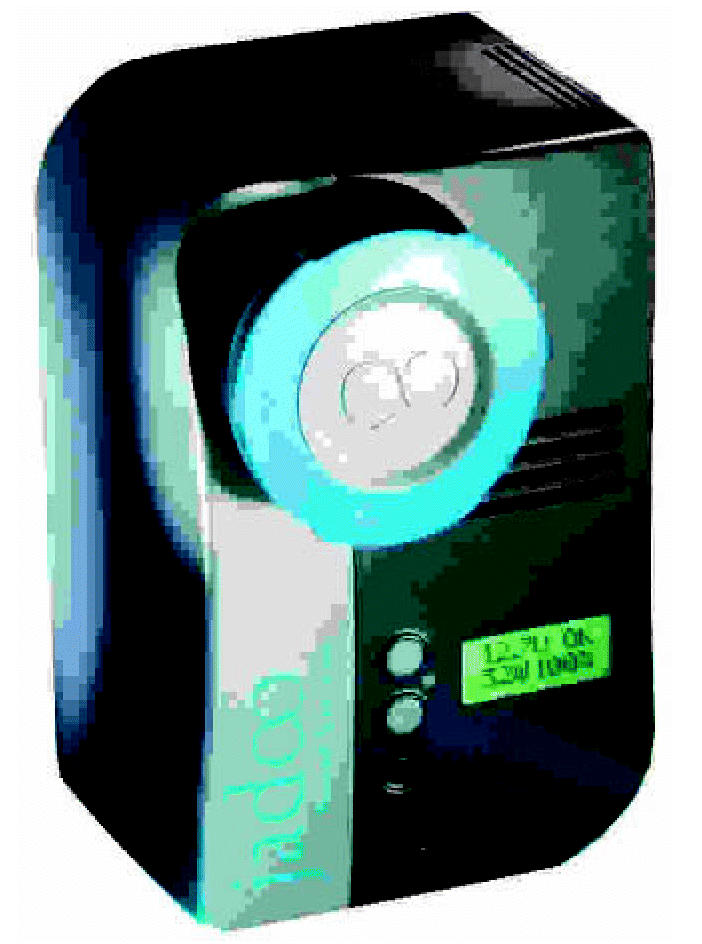


**Figure:**
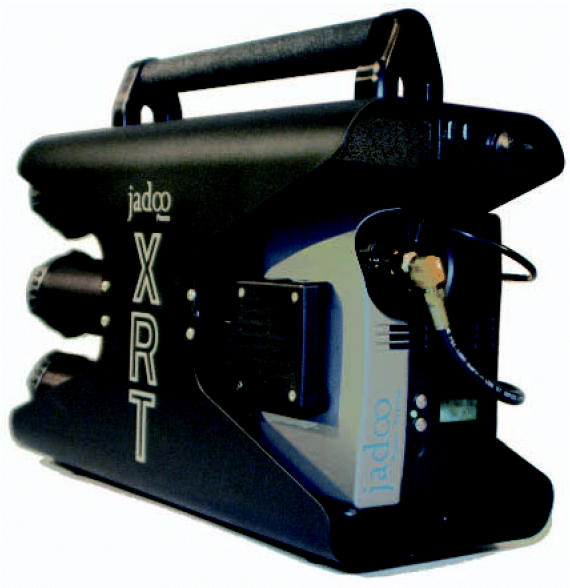
The XRT can power lighting, radios, phones. and more in emergencies.

**Figure:**
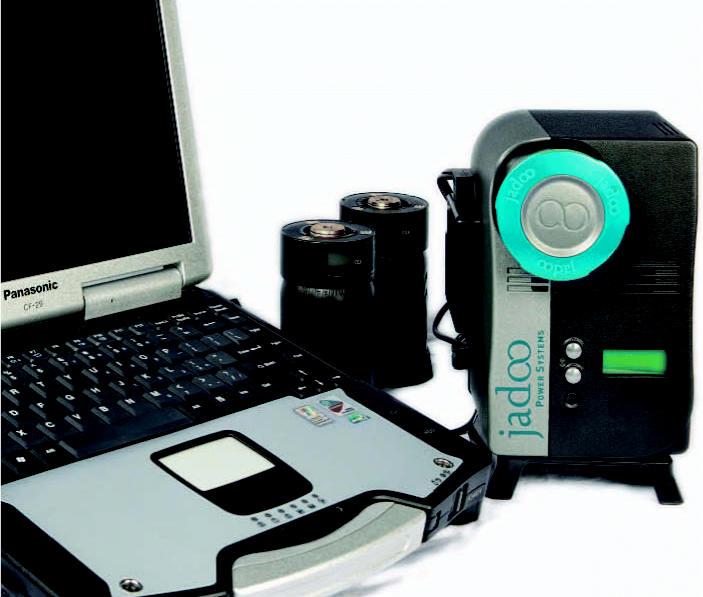
The Jadoo system could replace current laptop batteries.

**Figure:**
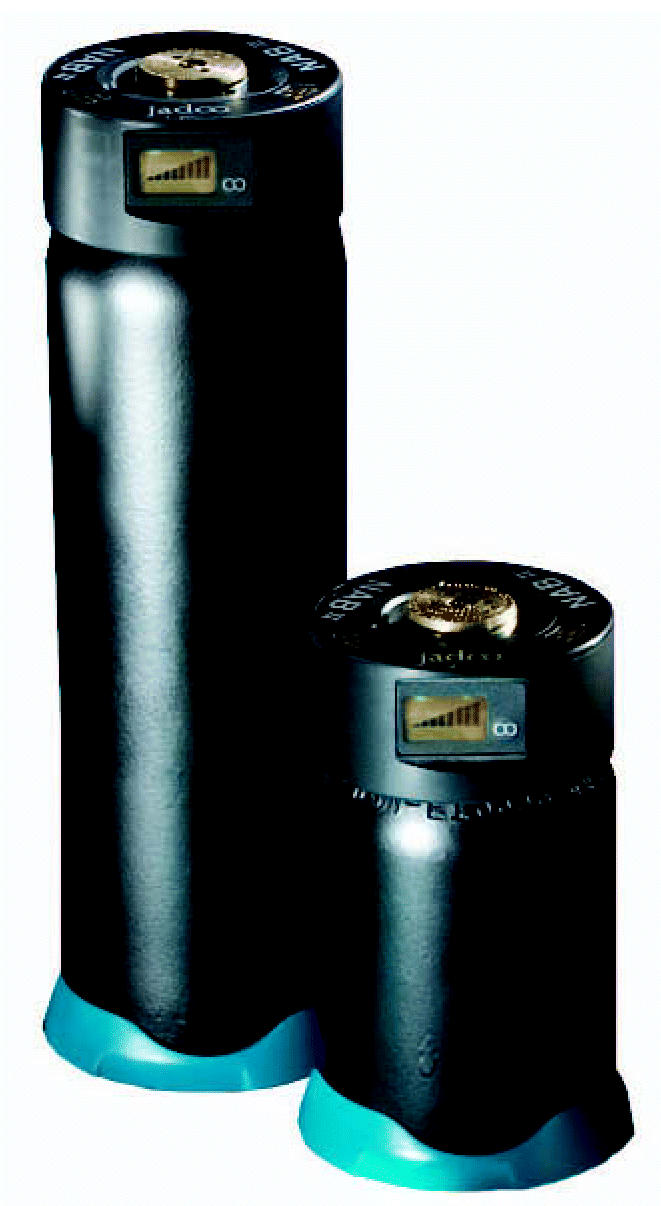
N-Stor canisters store the power.

**Figure:**
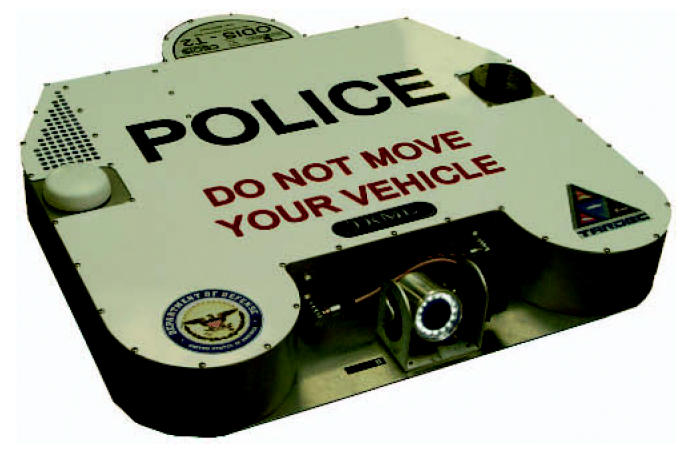
Potential military and police uses include under-car search robots.
